# Observations on the Treatment of Asthma

**Published:** 1861-08

**Authors:** T. L. Pridham

**Affiliations:** Bideford, North Devon


### SELECTIONS.

### *Observations on the Treatment of Asthma.* By T. L. Pridham, Esq., Bideford, North Devon.

Much has been written, of late, on the symptoms and treatment of Asthma—perhaps the most painful and apparently alarming disease to which human flesh is heir.

The observations which I have to make on the disease, are based on the experience of nearly one hundred cases which have come under my medical care.

I may not throw much new light on the subject; yet I am desirous to record the result of my experience and my treatment, as well as my observations on the peculiarities of this most distressing complaint, in the hope that some little may be added to the stock of information which has already been collected on the subject.

It is now many years since a deep-rooted impression was made on my mind by that memorable man, Abernethy, "that in order to treat disease, whether of a local or constitutional character, it was absolutely necessary to keep the stomach in that state, so that the food which was taken into it should be properly digested, in order to secure good blood, and consequently healthy secretions and healthy action of the body generally; and that in proportion as the surgeon or the physician attended to these particulars, so in proportion would the means placed within the reach of the practitioner be more or less successful."

With these impressions, which I believe to be most useful to the medical man, I started in my profession. I had not been long in practice, when a severe case of what was called humid asthma presented itself for my treatment. I found that it had been of many years standing, frequeutly seemed from its violence, to threaten the life of the patient. The attack however, would subside after copious expectoration had been thrown off from the lungs; the man, at the same time becoming almost exhausted for want of nourishment, which he was not for a time able to take, except in the smallest quantities. After the lapse of a few weeks, the man would rally, eat and drink largely, and go about his work until another paroxysm threatened, when I was again in attendance. On each occasion of attack, I used to say, "This surly will be the last," so severe and imminent did it appear.

This state of things went on for some time, till at last the thought occured to me, how is it that asthmatic patients generally live to a good old age? They do not die of consumption; nor do the lungs undergo any other change except the dilatation of the air-cells, which dilatation of the cells does not much interfere with their normal action when free from an attack. Thus consdering the matter, it appeared to me probable that the original cause of disease was in the overworked powers of digestion; and I was borne out in this supposition by the circumstance that the patient had respite from attacks as soon as a certain amount of expectoration had been thrown off, and his stomach had for a season rested. Was it not then probable that impurities in the blood were formed by imperfect digestion, which were thrown off by means of the lungs! How far my ideas were correct, the following statement of treatment will, I think, go far to prove.

I speak now of what is called *dyspeptic asthma*—a disease which I believe peculiarly hereditary; that is to say, when the disease has once made its appearance, those who inherit it will ever be liable to attacks from imprudent diet; for I am convinced that an asthmatic person can never with impunity eat and drink as other people do. I may here state that, out of the number of cases which I have attended, I found hereditary predispositions in nine out of ten. And if it be true that asthma is an hereditary disease, I will endeavor, by the evidence of cases which I intend to publish, to show that wherever it has existed in any member of a family for one, two, or even three generations back, exciting causes, such as imprudent diet, an attack of bronchitis, an attack of influenza, derangement of the liver, or any other functional derangement, atmospheric influences, peculiar odors, influences of the mind, either for joy or sorrow, may at any time or period of life bring to light this peculiar disease, which has hitherto been latent in the system. It is fortunate that stomach-derangement from imprudent diet, etc., is, as far as my experience goes, the most frequent exciting cause—consequently more under the control of medical treatment, provided the patient has resolution enough to second the efforts of the medical attendant in carrying out a strict system of diet and regularity; in fact, it may be said that the scales are thus held with disease and suffering on one side, and comparative health and comfort on the other.

Most of the other patients inherited gout, and in some instances both diseases were traced in the family; and in more than one instance where this was apparent, I found that there were alternate attacks of asthma and gout, which is, I think, an additional evidence that both diseases are hereditary, and may be considered blood-diseases. What the peculiar element in the blood may be which produces either asthma or gout, it is to be hoped scientific researches may one day decide.

To return to my first case. The man in question, possessing some good sense, although he gave himself to beer-drinking and gross eating, as he said, to get up his strength after an attack, after having heard my views of his case, replied, "Well, sir, your remarks appear reasonable enough, and I will endeavor to follow your advise, and not eat and drink so much." I wrote down a system of dietary and general management, which for a season he followed with advantage; after this, he became tired of the plan, and he again fell into intemperate habits, with an increase, if possible, of all his original fearful sufferings. He at last became reduced in circumstances, and was obliged to apply for parochial aid. I thought I had now a good opportunity of trying the effects of diet with medical treatment on this patient, and I persuaded him to go into the union house. There I took good care that he should not deviate from my prescribed treatment; for I placed him unker *lock and key*, ordering his food to be regularly given him, whilst he had a trustworthy attendant when he left his room for the purpose of air or exercise.

His appearance and symptoms at this time were as follows: His countenance bore the signs of great distress; his shoulders were elevated; the eyes protruded; he had no appetite; the stomach was greatly distended after eating; pulse 70, feeble, yet regular; the tongue was coated, with fissures in it. There was great emaciation, and inability to lie down in bed or to walk up an ascent. The secretions from the bowels were dark. The urine was loaded, showing an acid deposit.

After giving the patient an alterative pill and a saline aperient, I ordered him the following diet, which was to be regularly weighed out to him, and the hours of meals most strictly attended to. Breakfast at eight o'clock—half a pint of green tea or coffee, with a little cream, two ounces of dry stale bread; dinner at one o'clock—two ounces of fresh beef or mutton without fat or skin, two ounces of stale dry bread, or well boiled rice; three hours after dinner, half a pint of weak brandy and water, or toast water *ad libitum:* supper at seven o'clock—two ounces of meat, with two ounces of dry bread. He was not allowed to drink within one hour of his dinner or supper, or till three hours after; at other times, he was not limited. Open air exercise was ordered to be taken as soon as the office of digestion had been performed, but short of fatigue. In addition to this, I ordered him three grains of the extract of conium four times a day, at the hours of seven, twelve, five, and ten; the dose is to be gradually increased to five grains four times a day. Under this treatment, in a few days the whole of his distressing symptoms subsided. At the end of a month he was allowed three ounces of meat twice a day. At the end of two months, he had gained flesh and strength, so that he was able to follow his occupation as a carpenter, which he continued to do without intermission for some years, adhering strictly to the rules laid down for him, which he had now the good sense to adopt.

My views with regard to treatment in the case were simply these: not to give the stomach more to do than it could well accomplish; not to dilute the gastric juices by fluids before or soon after eating, but allow them to perform alone their proper functions; and to quiet the nerves of the stomach by means of a mild sedative, which prepared it for the due performance of the office of digestion. By this plan the stomach soon began to crave for food, and a longing for the hour for taking nourishment appeared—good evidence that the powers of digestion were recovering.

Is it not reasonable, then, to suppose that a moderate quantity of food, well digested, supplies the system with more pure and nutricious blood than a large quantity of ill digested food can possibly do? It appears to me not unreasonable that the poison, as it may be called, which is generated by food ill assimilated, finds its way into the circulation; and hence arises the misery of hereditary asthma, which nothing but a paroxysm, and generally a copious expectoration will relieve.

I now proceed to the further evidence of cases which have been treated by me under the sedative and strict dietary system; and in doing so, I think I cannot give stronger proof of its efficacy than by quoting communications from patients themselves, as evidence of the relief they have obtained, and the life of comparative ease and comfort to them after adopting the system. But, before I go further, I would remark on the absolute necessity of procuring the best prepared extracts, which form of medicine I have invariably used for the purpose required. I have more than once been disappointed in my treatment, when my prescriptions have been sent to ordinary druggists, with whom sufficient care has not been taken in this matter; therefore I strongly advise medical men to procure the extracts from, or send their prescriptions to, some known first-rate practical chemist, whose reputation greatly depends on the purity of his chemical preparations. Genuine extracts retain for a long time their distinctive odor, remain moist, and do not mildew. The sedative extracts which I have generally used are those of henbane, conium, belladonna, and stramonium, gradually increasing doses and frequently substituting one extract for another, as symptoms appear to demand.

It is not my intention to enter into the more scientific department as to the supposed causes and effects of this mysterious and capricious disease, so ably treated of in the pages of this journal by Dr. Hyde Salter, whose remarks I have read with much interest; my object is simply to record my remarks on the history of cases as reported by my patients, together with observations made by myself during my professional attendance, which may, I trust, lead to the mitigation of sufferings. My own opinion is, that it is impossible, from the present knowledge we possess, to come to the *real causes* of the disease in asthma; but still I trust practical observations may be valuable, inasmuch as they may lead to scientific researches, not only as regards the state of the blood in asthmatic patients, but also as regards the peculiar expectoration which is thrown off, through the medium of the lungs, in the different forms of the disease, which appears to me to be nature's effort for relief; for it is my strong impression, as I have before stated, that it is in the blood that the secret lies, as, the more healthy is the state of the powers of digestion, so in proportion is the freedom of the patient from disease in dyspeptic asthma. Then, again, as regards persons being affected in certain localities, whilst perfectly free in others, may not the peculiar state of the atmosphere and impurities to the blood in some districts, whilst in others a more congenial atmosphere will lessen, and in some instances entirely remove, the impurity which exists in the circulating fluid in those predisposed to the disease by hereditary causes? I have, apparently, seen several instances of this kind, which I will hereafter name.

I will now proceed to record the history of cases of dyspeptic asthma.

*Case* 2.—In the year 1855, I was requested to visit a clergyman, upwards of 70 years of age, from the North of England, who had been residing in this town for some weeks, for the benefit of his health. I had frequently remarked him in the streets; and not knowing the exact history of his case, I considered he was in the last stage of consumption, so emaciated, so feeble and distressed, did he appear. I think I never saw any one walking at large so emaciated. I learned from him, that he had been afflicted with asthma for ten years, that during that time his clerical duties had been suspended, and that, in fact, he was quite a martyr. In this time of his affliction, he had sought every possible advice, both in London and in the country, but without any material benefit or relief; the general opinion given him was, that he would never recover, and that he must bear his affliction as best he could. He was not able to lie down in bed; and, for years, every night he had anticipated death before the morning came, when, however, a copious heavy expectoration would with difficulty be thrown from the lungs, and as the day advanced he became somewhat relieved, was then dressed, and passed the remainder of the day in great discomfort. Having inquired into his mode of living, I was told pretty nearly as follows:—At six in the morning he had a cup of strong coffee; at nine he had tea or coffee, toast. eggs, or a chop; he lunched at one, generally on bread, cheese, and porter; he had a substantial dinner, with porter and wine, and a fair quantity of port wine after dinner; he had tea or coffee in the evening, as well as supper afterwards. My remark was, "And for all this amount of nourishment, you are as thin as any human being can well be." He replied, "I have been invariably told that, without such support, I cannot live a month; and I take it to make up for the large amount I expectorate in the morning." I examined his chest and found neither tubercular deposit nor any lesion of the lungs; but evidently, from the very clear sound emitted on percussion, the lungs were emphysematous, particularly under the right clavicle, whilst lower down a dull sound was emitted. In the morning, the pulse was seventy in a minute, but as night came on it rose to ninety or a hundred. His tongue had a very morbid coat—large, with fissures in it; his stomach was generally distended, and, though he ate so much, he had never any great desire for food; his bowels were irregular; the secretions dark; the urine was loaded with a pink deposit. At the conclusion of my first visit, I surprised him by saying, "You will considerably improve in health under the treatment which I intend to adopt, and, although your age is such, I should not be surprised at your one day being in harness again." His reply was, "That is utterly impossible; my disease has taken such hold on me for so many years." He further said, "he looked forward to nothing but death, and he often prayed to be released from his sufferings." I went on to remark, that if my treatment was to benefit him, seeing it was an extreme case of dyspeptic asthma, he must make up his mind to one thing, and unless it was fully carried out, he must not expect to derive any good result; that was, he must eat just one fourth what he was daily in the habit of taking, and, in addition, his food must be weighed, and taken as regularly as the clock struck. His reply was, "If that be your plan, you will soon shorten my days." I begged him to try it for one week, which he at length acceded to. I commenced my treatment on the 23d of April, by giving an alterative dose of blue-pill, and a saline aperient. I then ordered three grains of the extract of henbane four times a day, and the same system of dietary as prescribed in my first case, allowing him, in the form of animal food, only two ounces of it twice a day. The result was, that at the end of a week he was able to lie down in bed and sleep several hours; his morning expectoration had lessened considerably; his appetite improved; his tongue cleared; the distension at his stomach lessened; the urine was clear; his spirits lightened; and he could exert himself with much more ease. He was now fully satisfied that the plan had so far succeeded; but his great fear was that he should be starved, by reason of so small a quantity of food being allowed him. At the end of ten days, I substituted the extract of conium for that of henbane, ordering him four grains four times a day. From this time, he improved rapidly until he left the town to return home, on the 14th of May. On the 19th of June, he wrote me from London, where he was staying with some friends: "I cannot be sufficiently thankful for the comparative ease and comfort which, through God's blessing, and your skillful management, I have now enjoyed for the last six weeks. The weather has been most unpropitious, notwithstanding which, I have not entirely avoided the cold; my nose is rather 'stuffy' from its effects, but no symptoms of asthma have yet appeared, and I trust, by my strictly adhering to your regimen, I may be spared any return." On the 30th of June, he wrote, from his home in the North of England—"I find the air here much keener than in Biddeford or London, and it has come chiefly from the East. I cough a little in the morning, on first waking; but I have felt nothing like asthma. I have adhered very generally to your rules, with only the addition of more bread to my breakfast and tea, which you have allowed me. My secretions appear all right. I am, as you saw me, almost a skeleton; but I am in hopes, however, that the wholesome food which I get here will be beneficial." I may remark here that the months of May and June, in 1855, were unusually cold, the East winds generally prevailing. On the 12th of July, my patient wrote: "I am perfectly free from asthma; my appetite is very good, and I greatly desire that my daily allowance may be increased; and the number of my pills, I think, may be reduced." I now ordered five grains of the extract of conium three times a day, and increased his daily allowance of food a little, keeping strictly to the hours of taking it. On the 7th of December, my patient informed me: "Notwithstanding the severity of the cold, and the dampnese of the atmosphere, I am quite free from asthma. I now take my medicine only twice a day; it appears to have a wonderful effect. I am able to lie down in bed all night; and I sleep six or seven hours at a time, and feel no difficulty in breathing, and I trust this winter I shall be able to remain at home without a return of my fearful disease." On the 17th Dec., he wrote to say he was still going on well—"Some days since, I imprudently exposed myself too much to the cold and damp, and was consequently seized with a shivering fit, and afterwards fever. I was confined to my bed for forty eight hours; but no asthma made its appearance. I weighed myself last week; and notwithstanding my late attack, I found I had gained seven pounds in weight since the previous weighing."

Since the date of the last letter, I have occasionally heard from my patient, who still applies to me for advice whenever his complaint, from any imprudence in diet or irregularity, threatens an attack; and, to make my prediction true, I hear he has occasionally resumed his parochial duties as rector of his parish.

CASE III.—The third case of dyspeptic asthma was also in a clergyman, whose appearance was very different from that of the last, he being stout and lethargic. He came to be under my care from the county of Sussex, in 1856. He had been afflicted for seven years, and his difficulty of breathing was constantly on him whilst awake, particularly after a full meal. On examination, I found his lungs greatly oppressed, and dull on percussion; his hearts-action was very feeble and slow; his tongue coated and brown; the stomach greatly distended with flatus, which appeared to distress him greatly; the urine was loaded; he was unable to lie down in bed, and had heavy expectoration in the morning. He still performed his parochial duties, though with the greatest difficulty. Having cleared out the bowels by means of an aperient, I placed him on the sedative and strict dietary system, giving three grains of the extract of conium four times a day, with a pill every other night of two grains of each of the extracts of taraxacum and simple alœtic pill.

He came under my medical care on the 1st September. On the 8th of the same month, I had the following *bulletin* from him. "My health improves rapidly under your mode of treatment, and I was able yesterday to repeat the responses at church with ease to myself; and I took a walk without feeling the least oppressed in my breathing or fatigue to myself. I have been afflicted with asthma for more than seven years, and have consulted several eminent physicians without anything like the relief which I even now enjoy. A week only has elapsed since I consulted you; and yet, I am thankful to say, my chest is so much relieved, that I am able to walk several miles without being obliged to rest in order to fetch breath." On the 18th Sept., he wrote:—"By this time you will be anxious to know how I am going on. The weather has certainly been very fine and clear since I commenced your mode of treatment; but many a year has elapsed since I have had such freedom from pain. On Thursday last, I walked up a steep hill of half a mile without stopping once; and on the following day, I bore the fatigue of a long journey into Sussex, without finding my chest at all oppressed. Last Sunday was the grand criterion; and I can assure you my voice was remarkably clear, and my chest but scarcely influenced by the enemy." On the 2nd October, he wrote:—My health, on the whole, is very satisfactory. Since I last wrote you, the weather has been very cold and stormy; notwithstanding which, I have breathed more freely than I have for many a year, and I now go about the active duties of my calling with confidence."

Another letter, a month afterwards, tells me he is quite well; since which time I have had no communication with my patient, but I learn from his friends that, thinking that he had remained so long free from complaint, he might with impunity return to his former way of eating and drinking whatever came in his way, he has consequently since experienced returns of his complaint, as might naturally be expected; for, as I have before stated, an asthmatic patient, who inherits asthma, can never with impunity eat and drink as other people; and although I gradually increased the amount of support taken in this case, yet the patient was not satisfied, but allowed his inclination to overrule his better judgment.

The above case must be admitted to be strong proof how much the disease is influenced by the manner in which the office of digestion is performed, and how much the patient has it in his own power to live a life of comparative ease and comfort.—*British Med. Journal, June* 9 *and July* 28, 1860, *pp.* 434, 570.

---

*On the Essential Nature of Asthma.* By Dr. H. Hyde Salter, F. R. S.

There are two ways (which I have not mentioned in my work,) as indicated to me by my friend Dr. Brown-Sequard, in which bronchial spasm, when once established, may be kept up by the very conditions which it generates; one is, the power which carbonic acid gas possesses of producing contraction in smooth muscles. In asthma, the deficient standard at which respiration is carried on, and the diminished interchange of the gasses, produces an accumulation, in the air locked up in the air-passages, of carbonic acid to an unusual degree. This, by the action to which I have just referred, sets the bronchial muscles still further contracting, and thus increases the very condition which at first caused the accumulation of the effete gas. In this way, asthma keeps up asthma. The other way is by the bronchial spasm stimulating the afferent or perceptive nervous filaments, and thus giving rise to reflex muscular contraction; just as the stimulation of the sensitive roots of the spinal nerves produces reflex muscular phenomena in the parts to which the corresponding motor nerves are distributed. In this way, muscular spasm becomes a stimulus to muscular spasm.

But in both these, as in the other ways I have indicated in my book, the bronchial spasm is secondary to an antecedent nervous condition.—*British Med. Journal, July* 28, 1860, *p*, 589.

---

*On the Proper use of Stramonium in Hay-Asthma.* By Dr. EDWARD LAWFORD, Leighton Buzzard.

[The thorn-apple or stramonium was first introduced to our notice from the island of Ceylon. Authors differ remarkably in their testimony as to its use—some reporting favorably, others considering it well nigh valueless.]

Smoking the herb is the plan generally recommended, and in those who are easily brought under its influence this plan is, doubtless, occasionally of considerable service; but with the majority little or no effect is produced by this mode of procedure. True, smoking, if long continued, may produce nausea and slight narcotism; but the paroxysm is urgent, and *immediate* relief is required. Under these circumstances, I have been in the habit of directing the patient to introduce portions of the stem and leaf of the plant into his pipe, as before, to smoke it, and inject the smoke thus obtained from the mouth into an inverted ale glass; to continue this procedure until the glass is filled with the stramonium fumes, and then to place the glass (still inverted) over the mouth, and take one long and deep inspiration. The effect is instantaneous, a sense of suffocation for the moment, then copious expectoration of ropy mucus, and immediate relief.

Stramonium, in addition to its anti-spasmodic and narcotic properties, is a *direct, powerful and instantaneous expectorant,* if properly applied, and in this consists its peculiar value in this distressing complaint; but in order to effect this opject, it must be brought into *absolute contact* with the air-cells of the lung; simple imbibition through the mucus membrane of the mouth will not bring out its powerful expectorant properties, and hence those who have looked for benefit from smoking the drug simply, have been grievously disappointed, and have pronounced it valueless.—*British Medical Journal, Aug.* 18, 1860, *p.* 657.—*Braithwait's Retrospect.*

[THE FOLLOWING ARE FROM THE "SUMMARY OF MEDICAL SCIENCE.]

*Toothache of Pregnancy—Submucous Injection.* Dr. H. R. Storer mentions, in the "Dental Cosmos," Nov., 1859, the following case:

Patient had suffered for several wreks from severe neuralgic pain throughout the left half of the upper jaw, at times lancinating in its character, at others more dull, but never wholly absent.

The general health was decidedly affected, as evidenced by the state of the circulatory, digestive and nervous systems.

The teeth, on inspection, were all sound; there was no heat or swelling of the gums, no tenderness or increase of pain on pressing them

Anodynes, both local and general refrigerants, emollient poultices, and counter irritants were successively resorted to, without benefit.

After much solicitation, a tooth was extracted ; the patient remained unrelieved.

On the following day no change for the better having occurred, ten drops of the Edinburgh solution of bi-meconate of morphia were injected beneath the mucous membrane of the gum ; the pain ceased instantaneously, and from that moment to the present, a period of nearly five months, there has been no return of the malady.

